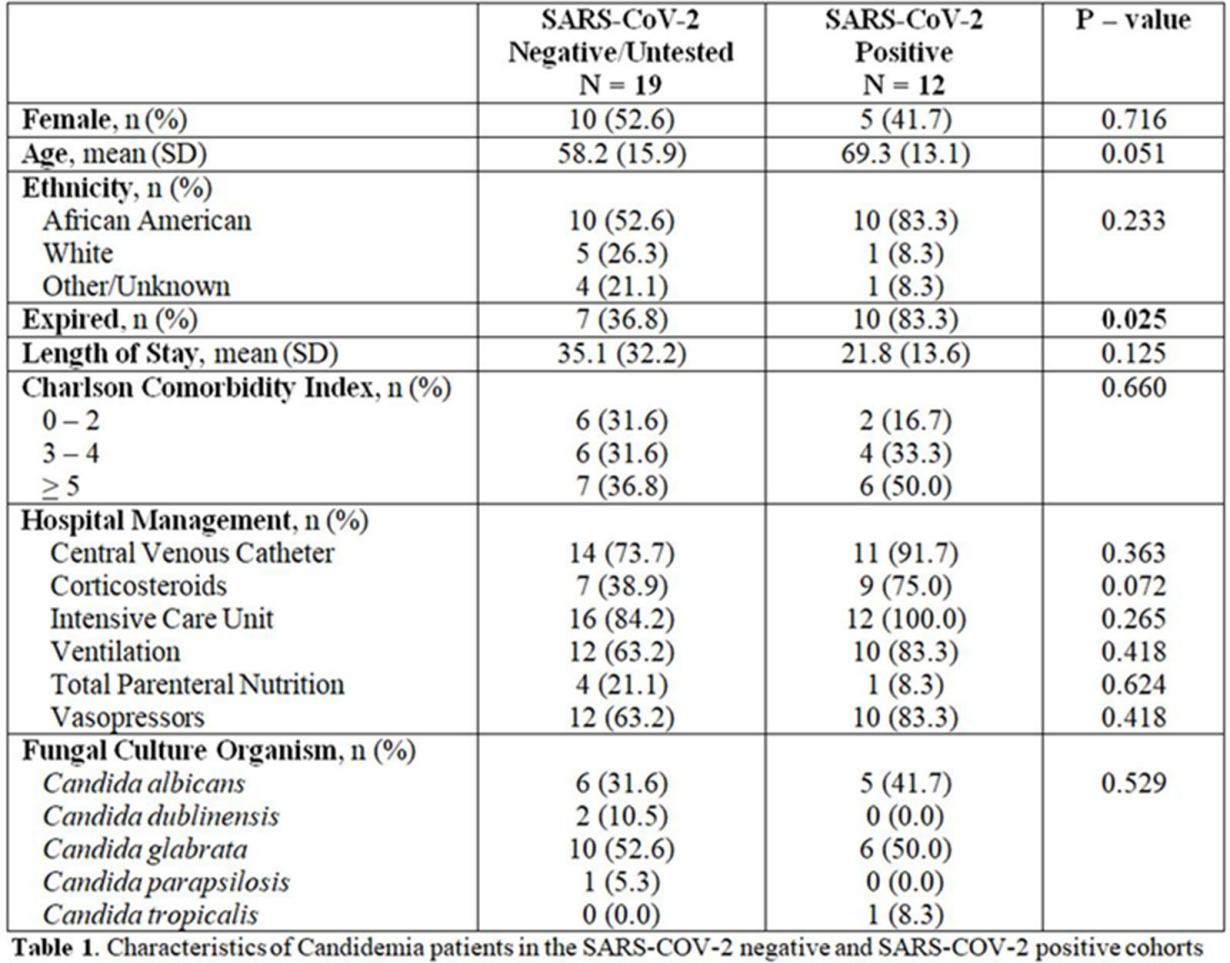# Comparison of Outcomes in Candidemia Between COVID-19 and Non–COVID-19 Patients

**DOI:** 10.1017/ash.2021.112

**Published:** 2021-07-29

**Authors:** Angela Beatriz Cruz, Jennifer LeRose, Teena Chopra

## Abstract

**Background:** Fungemia is associated with high rates of morbidity, mortality, and increase in length of hospital stay. Several studies have recognized increased rates of candidemia since the COVID-19 pandemic. **Methods:** Patients with candidemia during January through May 2020 were identified through Theradoc. Patient demographics, comorbidities, hospital management, and microbiology were extracted by medical chart review. Patients were divided into cohorts based on COVID-19 status. The Fisher exact and Satterthwaite tests were used for analyses of categorical and continuous variables, respectively. **Results:** Overall, 31 patients developed candidemia and 12 (38.7%) patients tested SARS-CoV-2 positive. *Candida glabrata* was the most prevalent causative organism in both groups. On average, COVID-19 patients developed fungemia 12.1 days from admission, compared to 17.8 days in the COVID-19 negative or untested cohort (*P* = .340). Additionally, COVID-19 patients with a fungemia coinfection were significantly more likely to expire; 10 COVID-19 patients (83.3%) died, compared to 7 (36.8%) in the COVID-19–negative or untested cohort (*P* = .025). The cohorts did not demonstrate statistically significant differences in terms of demographic, comorbidities, hospital management, or coinfections. **Conclusions:** The prevalence of fungemia in COVID-19 patients is significantly greater than historically reported figures. Known risk factors for candidemia, such as use of corticosteroid, use of central venous catheters, and prolonged ICU length of stay were higher in the SARS-CoV-2–positive cohort in this period, which likely contributed to increased fungemia rates, as these factors are also more pronounced in those with COVID-19. Patients who developed candidemia in the COVID-19 cohort had poorer outcomes than those who were SARS-CoV-2 negative or were untested. Further investigation should be conducted in larger studies.

**Funding:** No

**Disclosures:** None

**Table 1.**